# Alpha-synuclein co-pathology amplifies amyloid-driven tau accumulation across Braak stages without modifying tau-cognition associations

**DOI:** 10.64898/2026.03.31.713304

**Published:** 2026-04-06

**Authors:** Ahmed Negida

**Affiliations:** 1 Department of Neurology, Virginia Commonwealth University, Richmond, VA, USA; 2 Parkinson’s and Movement Disorders Center, Virginia Commonwealth University, Richmond, VA, USA

**Keywords:** alpha-synuclein, seed amplification assay, amyloid, tau, ATN framework, co-pathology, Braak staging, Alzheimer’s disease, ADNI, biomarkers

## Abstract

**INTRODUCTION:**

Alpha-synuclein (αSyn) is the most common co-pathology in Alzheimer’s disease (AD), yet its role within the amyloid–tau–neurodegeneration (ATN) cascade is unknown.

**METHODS:**

We analyzed 636 ADNI participants with CSF αSyn seed amplification assay, amyloid PET, regional tau PET (Braak I–VI), structural MRI, and cognitive composites. Interaction models tested whether αSyn modifies the amyloid–tau and tau–cognition associations.

**RESULTS:**

αSyn positivity (19.0%) amplified the amyloid–tau association across all Braak stages (meta-temporal interaction β = 0.258, 95% CI 0.104–0.411, p = 0.001), with strongest effects in Braak III–IV. αSyn did not modify tau–cognition associations in any domain (all interaction p > 0.18).

**DISCUSSION:**

αSyn co-pathology selectively amplifies amyloid-driven tau propagation without modifying downstream tau–cognition relationships, identifying a node-specific effect within the ATN cascade with implications for patient stratification.

## 1. Background

Clinicians who evaluate patients along the Alzheimer’s disease (AD) continuum recognize a striking paradox: two individuals with comparable amyloid burdens on PET can follow vastly different clinical trajectories. One may remain cognitively intact for years; the other deteriorates rapidly [1,25]. The NIA-AA amyloid–tau–neurodegeneration (ATN) framework posits a linear cascade—Aβ plaques drive tau hyperphosphorylation, neurofibrillary tangle spread, neurodegeneration, and cognitive decline [1]—yet this model alone cannot explain the clinical heterogeneity we observe at the bedside. Identifying the biological modifiers that accelerate or decelerate specific steps in this cascade is therefore a priority for both prognosis and therapeutic targeting.

Among candidate modifiers, alpha-synuclein (αSyn) co-pathology stands out for its frequency and biological plausibility. Autopsy series by Hamilton [2] and Irwin et al. [3] have consistently shown that 40–60% of pathologically confirmed AD brains harbor Lewy body inclusions. Critically, this co-occurrence is not merely a late-stage epiphenomenon. In transgenic models, Clinton et al. [4] demonstrated that αSyn, Aβ, and tau interact synergistically, accelerating neuropathology and cognitive decline beyond what any single proteinopathy produces. Guo et al. [5] later showed that distinct αSyn fibril strains can directly cross-seed tau aggregation in primary neurons and in vivo, while soluble αSyn oligomers have been found to interact with both Aβ and tau, modulating aggregation kinetics through cross-seeding mechanisms [6,24]. These preclinical observations raise a specific question for the clinician: does αSyn co-pathology modify how amyloid drives tau accumulation in human brain, and does it alter the downstream relationship between tau and cognition?

Until recently, this question could only be addressed at autopsy—by which time the interplay among proteinopathies reflects end-stage disease. The development of cerebrospinal fluid (CSF)-based seed amplification assays (SAA) has changed this. Siderowf et al. [16] validated the Amprion SAA in the Parkinson’s Progression Markers Initiative cohort, demonstrating high sensitivity and specificity for detecting αSyn aggregation in living individuals. When applied to the ADNI cohort, Tosun et al. [7] showed that αSyn SAA positivity was associated with amyloid pathology, faster cognitive decline, and earlier symptom onset—establishing that αSyn copathology is clinically relevant along the AD continuum, not only in synucleinopathies.

Building on this foundation, Franzmeier et al. [8] recently provided the first direct evidence that αSyn co-pathology accelerates amyloid-driven tau accumulation in AD. Using 592 participants with CSF αSyn SAA, CSF p-tau, and longitudinal tau PET, they showed that αSyn-positive individuals had steeper amyloid-to-tau slopes. However, their analysis focused on a composite tau measure and did not examine whether αSyn modifies the downstream tau-to-cognition step—a clinically critical question, because it determines whether αSyn status should alter how we interpret tau PET findings when counseling patients. Nor did they assess regional tau specificity across the full Braak staging hierarchy, which is needed to understand where in the cortex αSyn exerts its modulatory effect.

We designed this study to fill both gaps. In 636 ADNI participants with concurrent αSyn SAA, amyloid PET, regional tau PET across Braak stages I–II through VI, structural MRI, and domain-specific cognitive composites, we systematically tested two hypotheses: (1) αSyn co-pathology amplifies the amyloid-to-tau association across all Braak stages, and (2) αSyn co-pathology modifies the tau-to-cognition association across memory, executive function, language, and visuospatial domains.

## 2. Methods

### 2.1 Study design and participants

Data were obtained from the Alzheimer’s Disease Neuroimaging Initiative (ADNI) database (adni.loni.usc.edu). The ADNI was launched in 2003 as a public-private partnership, led by Principal Investigator Michael W. Weiner, MD. The primary goal of ADNI has been to test whether serial magnetic resonance imaging (MRI), positron emission tomography (PET), other biological markers, and clinical and neuropsychological assessment can be combined to measure the progression of mild cognitive impairment (MCI) and early Alzheimer’s disease (AD). For up-to-date information, see www.adni-info.org [26]. Participants included cognitively normal (CN), mild cognitive impairment (MCI), and dementia subjects enrolled in ADNI-1, ADNI-GO, ADNI-2, and ADNI-3. The study was approved by institutional review boards at each participating site, and written informed consent was obtained from all participants.

### 2.2 Inclusion and exclusion criteria

**Figure 1.**
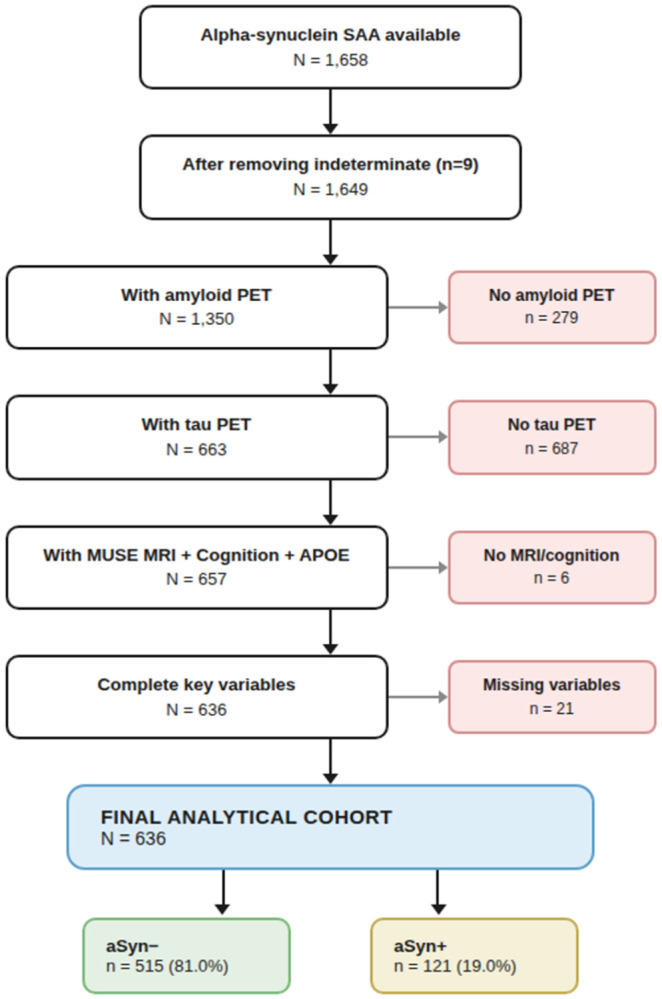
Study flow diagram. Of 1,658 ADNI participants with alpha-synuclein seed amplification assay (SAA) data, 636 met inclusion criteria. Abbreviations: αSyn, alpha-synuclein; PET, positron emission tomography; MRI, magnetic resonance imaging.

We included ADNI participants with available CSF-based αSyn SAA results and concurrent amyloid PET, tau PET (flortaucipir), structural T1-weighted MRI (processed through the MUSE pipeline), cognitive composite scores (PHC composites), and APOE genotype. Participants with indeterminate αSyn SAA results were excluded (n = 9). The exclusion cascade proceeded as follows: of 1,658 participants with αSyn SAA, 1,649 had determinate results (1,629 unique subjects); inner joining with amyloid PET yielded 1,350; further requiring tau PET reduced this to 663; adding MUSE MRI left 657; cognitive and APOE data were available for all 657; exclusion of 21 participants with missing key analysis variables produced the final analytical cohort of N = 636 (Figure 1).

### 2.3 Alpha-synuclein seed amplification assay

αSyn aggregation was assessed using the Amprion CSF-based SAA, which detects misfolded αSyn seeds through cyclic amplification of recombinant αSyn substrate [16]. Results were classified as Detected-1 (single replicate positive), Detected-2 (both replicates positive), Not Detected, or Indeterminate. Participants with Detected-1 or Detected-2 results were classified as αSyn-positive; all others as αSyn-negative. The earliest available SAA result per participant was used [7].

### 2.4 Amyloid PET

Amyloid PET was performed using florbetapir ([18F]AV-45), florbetaben ([18F]FBB), or Pittsburgh Compound B ([11C]PiB), with centiloid values computed through the ADNI Preclinical and Harmonized Composites (PHC) pipeline to enable cross-tracer comparability [17]. Amyloid positivity was defined using tracer-specific thresholds harmonized to a centiloid scale (≥20 centiloids). The earliest available scan per participant passing quality control was used.

### 2.5 Tau PET

Tau PET was performed using flortaucipir ([18F]AV-1451). Regional standardized uptake value ratios (SUVRs) were extracted from the ADNI PHC detailed tau dataset, which provides Braak stage-specific composites (Braak I–II, III, IV, V, VI) and a meta-temporal composite [18]. Tau positivity was defined as meta-temporal SUVR > 1.30, a validated threshold for detecting early temporal neocortical tau pathology [19]. Scans failing quality control were excluded.

### 2.6 Structural MRI and neurodegeneration markers

T1-weighted structural MRI scans were processed using the MUSE (Multi-atlas region Segmentation utilizing Ensembles) pipeline [20]. Hippocampal volume was calculated as the sum of left and right hippocampal MUSE ROIs (ROIs 47 and 48). The SPARE-AD (Spatial Pattern of Abnormality for Recognition of Early AD) score, an MRI-based machine-learning index of AD-typical neurodegeneration, was used to define neurodegeneration status (N+) using a cohort-median split [21]. Higher SPARE-AD values indicate more AD-like brain patterns.

### 2.7 Cognitive assessment

Cognitive performance was assessed using the ADNI PHC cognitive composite scores, which provide domain-specific measures for memory (PHC_MEM), executive function (PHC_EXF), language (PHC_LAN), and visuospatial processing (PHC_VSP) [22]. These composites are derived from multiple neuropsychological tests using item response theory and are optimized for sensitivity to change across the AD spectrum.

### 2.8 ATN classification

Participants were classified according to the NIA-AA ATN framework [1]. Amyloid (A) status was determined by centiloid-based amyloid PET positivity. Tau (T) status was defined by meta-temporal SUVR > 1.30. Neurodegeneration (N) status was defined by SPARE-AD score above the cohort median. This produced eight ATN profiles (A±, T±, N±) and four simplified AT groups (A−T−, A−T+, A+T−, A+T+).

### 2.9 Statistical analysis

Baseline characteristics were compared between αSyn-positive and αSyn-negative groups using Mann–Whitney U tests (continuous variables) and chi-square tests (categorical variables). αSyn positivity prevalence across AT groups was compared using the chi-square test of independence.

The primary analysis employed ordinary least squares (OLS) linear regression with interaction terms to test two hypotheses. First, whether αSyn modifies the amyloid–tau association: tau_z ~ centiloids_z × αSyn_pos + age_z + sex + education_z + APOE4. Six models were fit, one for each tau region (meta-temporal, Braak I–II, III, IV, V, VI). Second, whether αSyn modifies the tau–cognition association: cognition_z ~ meta-temporal_tau_z × αSyn_pos + centiloids_z + age_z + sex + education_z + APOE4. Four models were fit, one for each cognitive domain. All continuous predictors and outcomes were z-standardized. Significance was set at α = 0.05 (two-sided). Given the hypothesis-driven nature of the analysis, no correction for multiple comparisons was applied to the primary interaction tests [23].

Pre-specified sensitivity analyses included: (1) restriction to amyloid-positive participants; (2) stratification by clinical diagnosis (CN, MCI); (3) logistic regression models for AT group membership; and (4) αSyn main-effect models without the interaction term. All analyses were conducted in Python 3.11 using statsmodels 0.14, scipy 1.11, pandas 2.1, and numpy 1.26. Random seed was set to 2025 for reproducibility.

## 3. Results

### 3.1 Cohort characteristics

Of 1,658 ADNI participants with αSyn SAA data, 636 met inclusion criteria after requiring concurrent amyloid PET, tau PET, structural MRI, cognitive composites, and APOE genotyping (Figure 1). The analytical cohort comprised 353 CN (55.5%), 246 MCI (38.7%), and 37 dementia (5.8%) participants, with a mean age of 71.4 years (SD 7.0), 52.0% female, and mean education of 16.5 years (SD 2.4).

αSyn SAA positivity was detected in 121 participants (19.0%). Compared with αSyn-negative individuals, αSyn-positive participants were significantly older (73.0 vs. 71.0 years; p = 0.004), had higher amyloid burden (centiloids 36.1 vs. 24.6; p = 0.003), greater meta-temporal tau SUVR (1.4 vs. 1.3; p < 0.001), higher SPARE-AD scores (−0.1 vs. −0.4; p < 0.001), smaller hippocampal volume (7,099 vs. 7,352 mm^3^; p = 0.016), and lower memory composite scores (0.3 vs. 0.6; p < 0.001). APOE ε4 carrier frequency was higher among αSyn-positive individuals (47.9% vs. 37.1%; p = 0.036). There were proportionally more dementia cases in the αSyn-positive group (14.9% vs. 3.7%; p < 0.001) (Table 1).

### 3.2 Alpha-synuclein positivity across AT biomarker profiles

**Figure 2.**
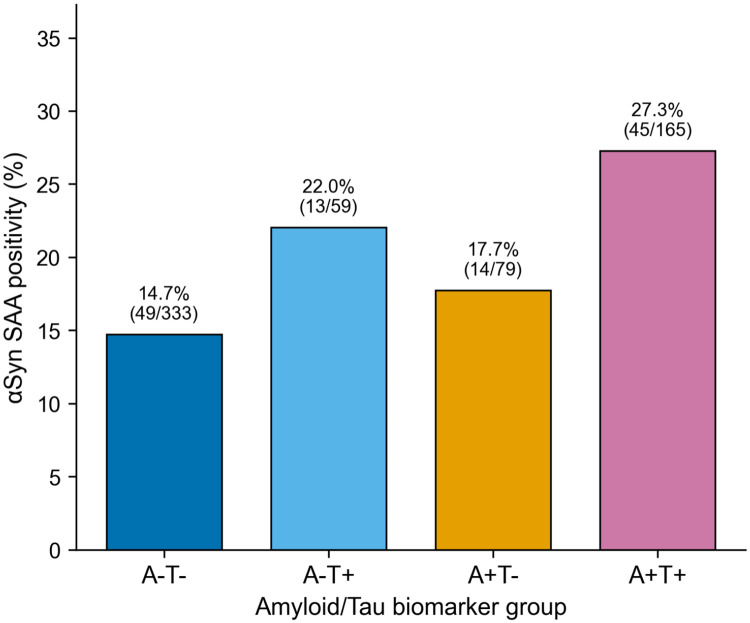
Alpha-synuclein SAA positivity prevalence across AT biomarker groups. Bars indicate the percentage of αSyn-positive participants. Numbers above bars show exact percentages and counts. χ^2^ = 11.74, p = 0.008.

αSyn SAA positivity prevalence differed significantly across AT biomarker groups (χ^2^ = 11.74, df = 3, p = 0.008). The highest prevalence was observed in the A+T+ group (27.3%; 45 of 165), followed by A−T+ (22.0%; 13 of 59), A+T− (17.7%; 14 of 79), and A−T− (14.7%; 49 of 333). Within the full ATN framework, the A+T+N+ subgroup had the highest αSyn positivity rate (33.1%; 40 of 121), whereas A−T−N− had the lowest (11.5%; 23 of 200) (Table 2, Figure 2).

### 3.3 Alpha-synuclein amplifies the amyloid–tau association

**Figure 3.**
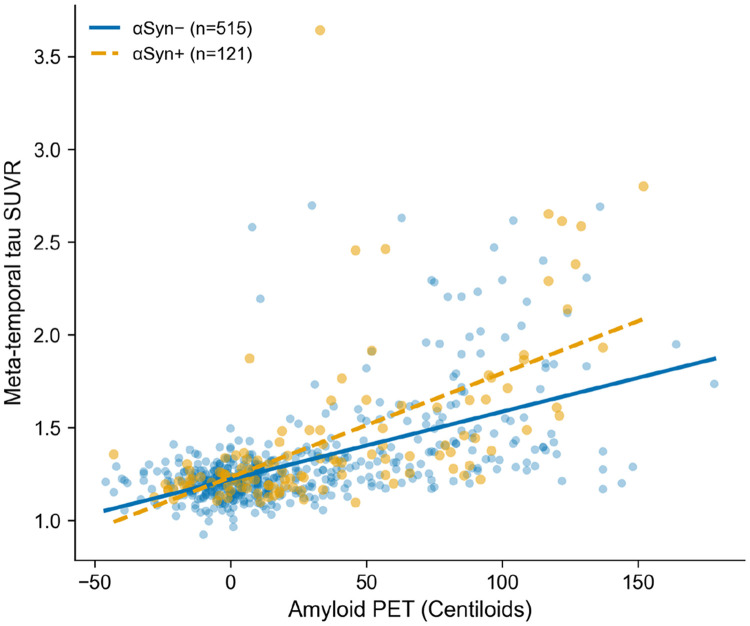
Amyloid burden versus meta-temporal tau PET uptake, stratified by αSyn SAA status. The steeper slope in αSyn+ participants reflects the significant interaction (β = 0.258, p = 0.001).

In multivariable linear regression models adjusted for age, sex, education, and APOE ε4 status, amyloid burden (centiloids) was strongly associated with tau PET uptake across all regions (all p < 0.0001). αSyn SAA positivity significantly amplified this association: the αSyn × centiloids interaction was significant for meta-temporal tau (β = 0.258, 95% CI 0.104–0.411, p = 0.001), Braak III (β = 0.250, 95% CI 0.095–0.404, p = 0.002), Braak IV (β = 0.264, 95% CI 0.107–0.420, p = 0.001), Braak V (β = 0.221, 95% CI 0.062–0.380, p = 0.007), Braak I–II (β = 0.176, 95% CI 0.030–0.322, p = 0.018), and Braak VI (β = 0.177, 95% CI 0.008–0.345, p = 0.040) (Table 3, Figure 3). The strongest interaction effects were observed in Braak III–IV regions.

### 3.4 Alpha-synuclein does not modify the tau–cognition association

**Figure 4.**
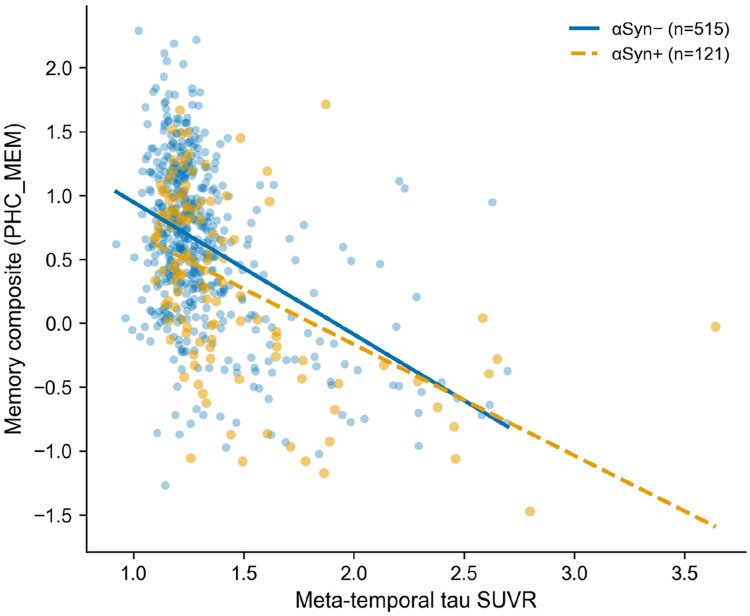
Meta-temporal tau versus memory composite, stratified by αSyn status. Near-parallel slopes confirm no αSyn × tau interaction on memory (β = −0.038, p = 0.578). The vertical offset reflects the additive αSyn effect.

In contrast to the amyloid–tau relationship, αSyn SAA positivity did not modify the association between tau burden and cognitive performance in any domain. Meta-temporal tau was strongly associated with all cognitive composites (memory: β = −0.331, p < 0.0001; executive function: β = −0.321, p < 0.0001; language: β = −0.323, p < 0.0001; visuospatial: β = −0.270, p < 0.0001). However, the αSyn × tau interaction term was non-significant for memory (β = −0.038, 95% CI −0.170 to 0.095, p = 0.578), executive function (β = 0.079, 95% CI −0.065 to 0.224, p = 0.283), language (β = 0.028, 95% CI −0.120 to 0.176, p = 0.712), and visuospatial function (β = 0.108, 95% CI −0.051 to 0.267, p = 0.183) (Table 4, Figure 4). αSyn positivity was independently associated with lower memory (β = −0.196, p = 0.019) and executive function (β = −0.231, p = 0.012) scores, suggesting an additive rather than synergistic effect on cognition.

### 3.5 Summary of interaction effects

**Figure 5.**
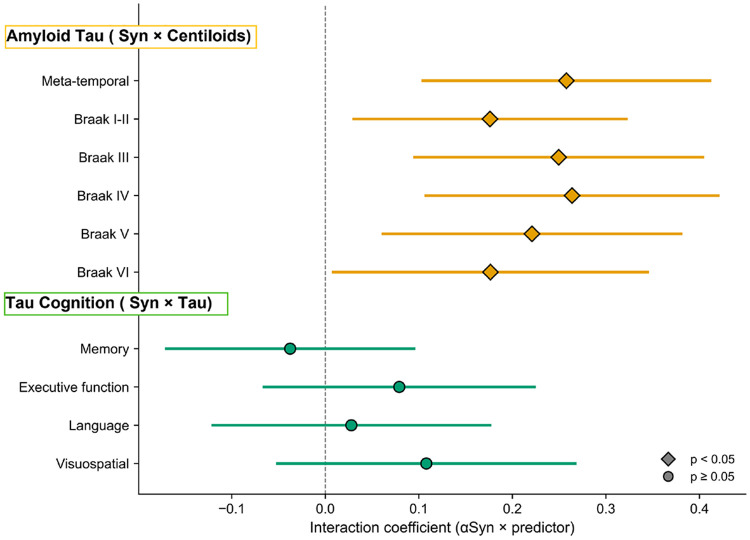
Forest plot of αSyn interaction effects across the ATN cascade. Upper: αSyn × centiloids on tau (amyloid–tau step). Lower: αSyn × tau on cognition (tau–cognition step). Diamonds: p < 0.05; circles: p ≥ 0.05. Error bars: 95% CI.

**Figure 6.**
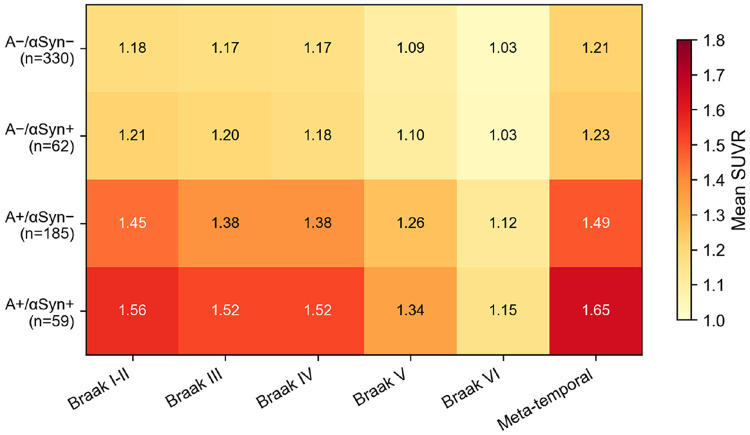
Regional tau PET SUVR heatmap by amyloid × αSyn status. Cell values represent mean SUVR. The A+/αSyn+ group shows the highest tau across all Braak regions.

Figure 5 summarizes the αSyn interaction effects across both cascade steps. All six amyloid-to-tau interaction coefficients are positive and significant, whereas all four tau-to-cognition coefficients are centered on zero and non-significant. The regional tau heatmap (Figure 6) shows that the A+/αSyn+ subgroup had the highest mean tau SUVR across all Braak stages, exceeding even the A+/αSyn− group, whereas in amyloid-negative individuals tau burden was uniformly low regardless of αSyn status.

### 3.6 Sensitivity analyses

We conducted four pre-specified sensitivity analyses. First, restricting to amyloid-positive participants (n = 244), the αSyn × centiloids interaction coefficients remained in the same direction (meta-temporal β = 0.270) but did not reach significance (p = 0.211), likely due to reduced sample size and range restriction. Second, diagnosis-stratified analyses in CN (n = 353) and MCI (n = 246) subgroups showed attenuated, non-significant interactions (CN meta-temporal β = 0.046, p = 0.638; MCI β = 0.110, p = 0.471). Third, logistic regression models showed that αSyn positivity was most strongly associated with the A+T+ profile (OR = 1.51, 95% CI 0.90–2.54, p = 0.118). Fourth, αSyn main-effect models confirmed independent associations between αSyn positivity and higher tau across all regions (meta-temporal β = 0.278, p < 0.001; Braak III β = 0.304, p < 0.001).

## 4. Discussion

We set out to determine whether αSyn co-pathology modifies specific steps of the ATN cascade in living individuals—a question that autopsy studies have raised but could never answer with temporal or regional precision. Our data from 636 ADNI participants reveal a clear dissociation: αSyn SAA positivity amplifies the association between amyloid burden and tau PET uptake across every Braak stage, yet leaves the downstream relationship between tau and cognition unchanged. In clinical terms, αSyn appears to act as an accelerant of tau propagation, not a blanket worsener of the entire cascade.

The most direct comparison for our findings is the recent work by Franzmeier et al. [8], who studied 592 participants with CSF αSyn SAA, CSF p-tau181, and longitudinal tau PET. They reported that αSyn-positive individuals showed steeper amyloid-to-tau slopes over time—an observation we independently replicate here using a different cohort, amyloid PET rather than CSF Aβ42/40, and regional tau PET across six Braak composite regions rather than a single composite. Our regional analysis adds an important dimension: the interaction is not uniform. The largest effect sizes appear in Braak III–IV (lateral temporal and fusiform cortex), which corresponds precisely to the stage at which tau first propagates beyond the medial temporal lobe [9,28]. This regional gradient was not examined by Franzmeier et al. and suggests that αSyn may be particularly relevant at the neocortical "bottleneck" where tau transitions from a localized to a diffuse pathology [12]. The strongest interaction in Braak III–IV also aligns with autopsy data from Irwin et al. [3], who found that AD brains with Lewy body co-pathology had more extensive neocortical tau than those without.

From the standpoint of biological mechanism, several lines of preclinical evidence converge on a model in which αSyn lowers the threshold for amyloid-driven tau misfolding. Guo et al. [5] showed that distinct αSyn fibril strains can directly cross-seed tau aggregation—both in primary neurons and in transgenic mice—establishing that the interaction is not merely associative but mechanistic. Vasconcelos et al. [10] extended this by demonstrating that Aβ-seeded tau fibrils become potent seeds for prion-like tau propagation in vivo, a process that αSyn co-pathology could amplify by providing additional misfolded protein templates. At the molecular level, soluble αSyn oligomers interact with both Aβ and tau through cross-seeding mechanisms [6,24], and McAllister et al. [11] have reviewed how all three proteins share prion-like propagation pathways that could synergize in the human brain. Clinton et al. [4] provided perhaps the most compelling in vivo evidence: triple-transgenic mice harboring Aβ, tau, and αSyn showed accelerated neuropathology and cognitive decline beyond what any single or double proteinopathy produced. Our human imaging data are consistent with this synergistic model.

Equally important is what we did not find. The absence of an αSyn × tau interaction on cognition across all four domains (memory, executive function, language, visuospatial processing) was consistent and unambiguous (all p > 0.18). This null finding is clinically meaningful: it tells us that once tau has accumulated in the cortex, the cognitive damage it inflicts proceeds at a rate that is independent of whether αSyn is present. For the clinician interpreting a tau PET scan, this means that tau burden retains its prognostic value regardless of αSyn status—a reassuring message for biomarker-guided prognostication. We note, however, that the main-effect models did show independent associations between αSyn positivity and lower memory and executive function scores, suggesting that αSyn may impair cognition through pathways separate from tau—perhaps through direct synaptic toxicity, dopaminergic dysfunction, or cortical Lewy body formation [13]. This is consistent with observations from the DLB Consortium by Coughlin et al. [29], who reported that αSyn SAA-positive individuals with AD co-pathology experienced faster cognitive and functional decline than those without, pointing to additive but mechanistically distinct contributions of each proteinopathy.

From a therapeutic perspective, these findings sharpen how we think about trial design in an era of anti-amyloid therapies. The CLARITY AD trial demonstrated that lecanemab slows cognitive decline in early AD [27], but treatment response varied substantially. Our data suggest that αSyn status may be one source of this variance: if αSyn co-pathology accelerates how quickly amyloid drives tau propagation, then αSyn-positive individuals may derive greater benefit from early amyloid clearance—before the tau cascade has progressed beyond a reversible stage. Stratifying trial populations by αSyn SAA, alongside ATN biomarkers, could improve both the precision of enrollment and our ability to detect treatment effects [14]. Tosun et al. [7] showed in this same ADNI cohort that αSyn SAA positivity predicted faster cognitive decline and earlier symptom onset along the AD continuum, further supporting the notion that αSyn status captures clinically relevant biological heterogeneity that current ATN staging alone misses.

We must acknowledge several alternative explanations. First, our cross-sectional design cannot establish directionality: it is possible that αSyn does not cause faster tau accumulation but instead co-occurs preferentially in individuals who are already on an aggressive amyloid-to-tau trajectory for other reasons. Disentangling this will require longitudinal studies with serial αSyn SAA and tau PET—a design that ADNI-3 is beginning to enable [26]. Second, αSyn SAA positivity may be a proxy for broader cellular dysfunction—neuroinflammation, lysosomal failure, or generalized proteostatic collapse—rather than reflecting a specific αSyn-mediated mechanism. Third, we cannot exclude confounding by unmeasured co-pathologies, particularly TDP-43 [15] and cerebrovascular disease, both of which are common in the ADNI age range and interact with the ATN cascade in ways that remain poorly understood.

Several limitations deserve explicit mention. Our analytical cohort was derived through inner joins requiring concurrent availability of αSyn SAA, amyloid PET, tau PET, MRI, and cognitive data, which may introduce selection bias toward participants who are healthier, more compliant, or more accessible to research centers. The sensitivity analysis restricting to amyloid-positive participants (n = 244) showed consistent point estimates but did not reach significance (β = 0.270, p = 0.211), likely reflecting reduced power from range restriction rather than a true null; however, we cannot rule out the latter. The dementia subgroup was small (n = 37), precluding meaningful subgroup analysis in the group where clinical implications are most immediate. Finally, the ADNI cohort is predominantly non-Hispanic White and well-educated, and the prevalence and clinical impact of αSyn co-pathology may differ in more diverse populations.

In summary, our findings identify a specific and clinically actionable node at which αSyn intersects the Alzheimer’s disease cascade: the amyloid-to-tau transition. αSyn co-pathology amplifies this step across all Braak stages—most strongly in the lateral temporal and fusiform regions where tau first breaches the neocortex—while leaving the tau-to-cognition relationship intact. These data argue against viewing αSyn as a non-specific disease accelerator and instead position it as a selective modifier of tau propagation, with direct implications for biomarker interpretation, patient stratification, and the design of combination therapies that target amyloid and αSyn in concert.

## Figures and Tables

**Table 1.** Baseline characteristics stratified by alpha-synuclein SAA status.

Characteristic	αSyn− (n = 515)	αSyn+ (n = 121)	p-value
Age, years	71.0 (7.1)	73.0 (6.2)	0.004
Female sex, n (%)	276 (53.6%)	55 (45.5%)	0.131
Education, years	16.5 (2.4)	16.6 (2.5)	0.733
APOE ε4 carrier, n (%)	191 (37.1%)	58 (47.9%)	0.036
Diagnosis			<0.001
CN	297 (57.7%)	56 (46.3%)	
MCI	199 (38.6%)	47 (38.8%)	
Dementia	19 (3.7%)	18 (14.9%)	
Amyloid PET (centiloids)	24.6 (41.2)	36.1 (44.7)	0.003
Amyloid positive, n (%)	185 (35.9%)	59 (48.8%)	0.012
Meta-temporal tau SUVR	1.3 (0.3)	1.4 (0.4)	<0.001
Braak I–II tau SUVR	1.3 (0.2)	1.4 (0.3)	<0.001
Braak III tau SUVR	1.2 (0.2)	1.4 (0.3)	<0.001
Braak IV tau SUVR	1.2 (0.2)	1.3 (0.4)	0.006
Braak V tau SUVR	1.2 (0.2)	1.2 (0.3)	0.029
Braak VI tau SUVR	1.1 (0.1)	1.1 (0.2)	0.155
Tau positive, n (%)	166 (32.2%)	58 (47.9%)	0.002
SPARE-AD score	−0.4 (0.5)	−0.1 (0.6)	<0.001
Hippocampal volume, mm^3^	7,352 (845)	7,099 (1,038)	0.016
Memory composite	0.6 (0.6)	0.3 (0.7)	<0.001
Executive function	0.7 (0.6)	0.4 (0.8)	0.001
Language	0.7 (0.6)	0.5 (0.6)	0.008
Visuospatial	0.4 (0.6)	0.3 (0.7)	0.103

Continuous variables: mean (SD); categorical: n (%). p-values from Mann–Whitney U (continuous) or χ^2^ (categorical). Tau positivity: meta-temporal SUVR > 1.30. Abbreviations: CN, cognitively normal; MCI, mild cognitive impairment; SUVR, standardized uptake value ratio; SPARE-AD, Spatial Pattern of Abnormality for Recognition of Early AD.

**Table 2.** Alpha-synuclein SAA positivity across ATN biomarker profiles.

ATN Profile	αSyn−	αSyn+	Total	αSyn+ (%)
A+T+N+	81	40	121	33.1
A+T+N−	39	5	44	11.4
A+T−N+	27	8	35	22.9
A+T−N−	38	6	44	13.6
A−T+N+	21	6	27	22.2
A−T+N−	25	7	32	21.9
A−T−N+	107	26	133	19.5
A−T−N−	177	23	200	11.5
All	515	121	636	19.0

A, amyloid status; T, tau status (meta-temporal SUVR > 1.30); N, neurodegeneration status (SPARE-AD > median). χ^2^ across AT groups: 11.74, df = 3, p = 0.008.

**Table 3.** Alpha-synuclein modifies the amyloid–tau association: interaction analysis.

Tau region	β amyloid	p	β αSyn	p	β interaction	95% CI	p	Adj. R^2^
Meta-temporal	0.514	<0.0001	0.234	0.005	0.258	0.104, 0.411	0.001	0.355
Braak I–II	0.551	<0.0001	0.230	0.004	0.176	0.030, 0.322	0.018	0.419
Braak III	0.510	<0.0001	0.262	0.002	0.250	0.095, 0.404	0.002	0.350
Braak IV	0.494	<0.0001	0.231	0.007	0.264	0.107, 0.420	0.001	0.331
Braak V	0.488	<0.0001	0.191	0.028	0.221	0.062, 0.380	0.007	0.304
Braak VI	0.441	<0.0001	0.162	0.077	0.177	0.008, 0.345	0.040	0.227

Standardized β from OLS: tau_z ~ centiloids_z × αSyn_pos + age_z + sex + education_z + APOE4. N = 636. β interaction: additional amyloid–tau effect in αSyn+ vs. αSyn− participants. CI, confidence interval.

**Table 4.** Alpha-synuclein does not modify the tau–cognition association.

Domain	β tau	p	β αSyn	p	β interaction	95% CI	p	Adj. R^2^
Memory	−0.331	<0.0001	−0.196	0.019	−0.038	−0.170, 0.095	0.578	0.363
Executive function	−0.321	<0.0001	−0.231	0.012	0.079	−0.065, 0.224	0.283	0.241
Language	−0.323	<0.0001	−0.141	0.131	0.028	−0.120, 0.176	0.712	0.203
Visuospatial	−0.270	<0.0001	−0.094	0.351	0.108	−0.051, 0.267	0.183	0.080

Standardized β from OLS: cognition_z ~ tau_z × αSyn_pos + centiloids_z + age_z + sex + education_z + APOE4. N = 636. Non significant interaction terms indicate that the tau–cognition slope does not differ by αSyn status.

## Research in Context

### Systematic review:

We searched PubMed for studies combining α-synuclein seed amplification assays with amyloid and tau PET in Alzheimer’s disease. One recent study (Franzmeier et al., 2025) demonstrated that α-synuclein co-pathology accelerates amyloid-driven tau accumulation. No study has examined whether α-synuclein modifies the downstream tau–cognition relationship or assessed regional tau specificity across all Braak stages.

### Interpretation:

In 636 ADNI participants, α-synuclein co-pathology amplified the amyloid–tau association across all Braak stages but did not modify tau–cognition relationships. This dissociation identifies α-synuclein as a node-specific modifier of the ATN cascade, acting at the amyloid-to-tau transition.

### Future directions:

Longitudinal studies with serial tau PET and α-synuclein SAA are needed to establish temporality. Clinical trials should evaluate whether α-synuclein stratification improves prediction of anti-amyloid treatment response.
